# Metabolic Signatures in Adipose Tissue Linking Lipophilic Persistent Organic Pollutant Mixtures to Blood Pressure Five Years After Bariatric Surgery Among Adolescents

**DOI:** 10.1021/acs.est.4c13902

**Published:** 2025-02-25

**Authors:** Shudi Pan, Zhenjiang Li, Douglas I. Walker, Brittney O. Baumert, Hongxu Wang, Jesse A. Goodrich, Sarah Rock, Thomas H. Inge, Todd M. Jenkins, Stephanie Sisley, Scott M. Bartell, Stavra Xanthakos, Xiangping Lin, Brooklynn McNeil, Anna R. Robuck, Catherine E. Mullins, Michele A. La Merill, Erika Garcia, Max T. Aung, Sandrah P. Eckel, Rob McConnell, David V. Conti, Justin R. Ryder, Lida Chatzi

**Affiliations:** † Department of Population and Public Health Sciences, Keck School of Medicine, University of Southern California, Los Angeles, California 90032, United States; ‡ Gangarosa Department of Environmental Health, Emory University, Atlanta, Georgia 30322, United States; § Department of Surgery, Northwestern University Feinberg School of Medicine and Ann & Robert H. Lurie Children’s Hospital of Chicago, Chicago, Illinois 60611, United States; ∥ Department of Pediatrics, University of Cincinnati College of Medicine, Division of Biostatistics & Epidemiology, Cincinnati Children’s Hospital Medical Center, Cincinnati, Ohio 45229, United States; ⊥ Department of Pediatrics, Baylor College of Medicine, USDA/ARS Children’s Nutrition Research Center, Houston, Texas 77030, United States; # Department of Environmental and Occupational Health, Department of Epidemiology and Biostatistics, and Department of Statistics, University of California, Irvine, California 92697, United States; ¶ Division of Gastroenterology, Hepatology, Nutrition, Cincinnati Children’s Hospital Medical Center, Department of Pediatrics, University of Cincinnati College of Medicine, Cincinnati, Ohio 45229, United States; ∇ Department of Genetics, Stanford University School of Medicine, Stanford, California 94305, United States; ○ Irving Institute for Clinical and Translational Research, Columbia University, New York, New York 10027, United States; ⧫ Department of Environmental Medicine and Public Health, Icahn School of Medicine at Mount Sinai, New York, New York 10029, United States; †† Department of Environmental Toxicology, University of California, Davis, California 95616, United States

**Keywords:** adipose tissue, high-resolution metabolomics, mixture analysis, environmental chemical mixtures, hypertension, metabolome-wide association study

## Abstract

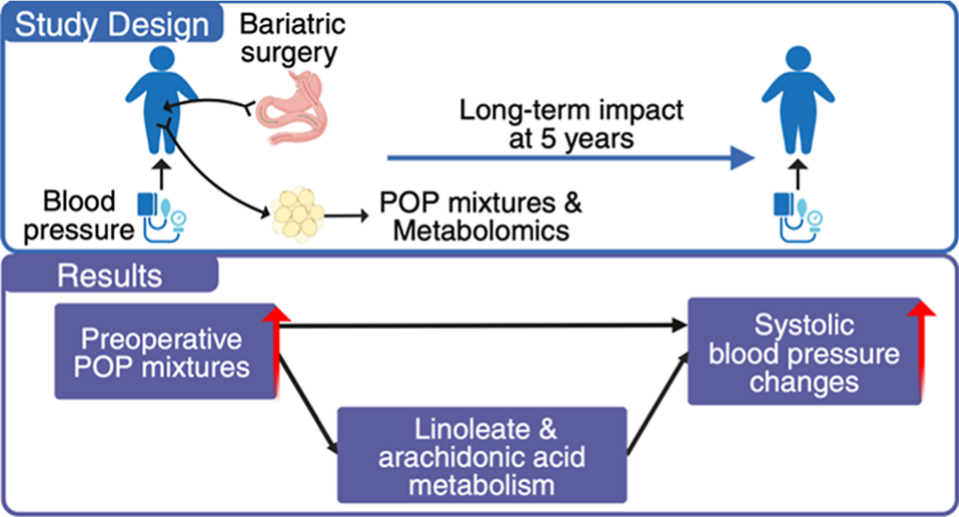

Persistent organic pollutants (POPs) are lipophilic environmental contaminants accumulated in the adipose tissue. Weight loss interventions, such as bariatric surgery, can mobilize POPs from adipose tissue into the bloodstream. We hypothesized that this mobilization could contribute to increases in blood pressure among 57 adolescents with severe obesity undergoing bariatric surgery. POPs and metabolic features were measured from visceral adipose tissue collected during surgery using gas and liquid chromatography, coupled with high-resolution mass spectrometry. Blood pressure was assessed at baseline, 6 months, and 5 years post-surgery. We used quantile g-computation to estimate associations of POP mixtures with blood pressure changes. With one quartile increase in POP mixtures, systolic blood pressure (SBP) increased by 6.4% five years after bariatric surgery compared to baseline SBP [95% confidence interval (CI): 0.4%, 12.4%]. The meet-in-the-middle approach identified overlapping metabolic features and pathways linking POP mixtures to SBP changes, highlighting the role of prostaglandin formation via arachidonic acid metabolism. POP mixtures were negatively associated with indole-3-acetate (−0.729, 95% CI: −1.234, −0.223), which was negatively associated with SBP changes at five years (−3.49%, 95% CI: −6.51%, −0.48%). Our findings suggested that lipophilic POP mixtures attenuated the beneficial effect of bariatric surgery on improved blood pressure among adolescents via alterations in lipid metabolism.

## Abstract 2

Exposures to persistent organic pollutants may promote blood pressure elevation through the lipid metabolism.

## 1 Introduction

Cardiovascular disease (CVD) is the leading cause of death in the United States and worldwide, with hypertension as a major modifiable risk factor.^1^ Hypertension in adolescence is associated with increased risks of CVD and CVD-related mortality in adulthood, independent of post-adolescent blood pressure levels.^2,3^ Intervening in early life hypertension is believed to be an effective strategy to reduce the risk of CVD. Persistent organic pollutants (POPs) have emerged as significant modifiable risk factors for hypertension, likely due to their roles in promoting inflammation, impairing vascular function, or disrupting the endocrine system.^4^ POPs are synthetic chemicals used in manufacturing products, such as flame retardants and pesticides. Despite efforts to ban their use, such as the 2001 Stockholm Convention, POPs remain as a global public health challenge.^5^ POPs have been detected at concerningly high levels worldwide, including in regions where these chemicals were never manufactured or used.^6^ Due to their long half-lives in environment, the general population continues to be exposed to POPs through diet.^7^ Lipophilic POPs, such as organochlorine pesticides (OCP), polychlorinated biphenyls (PCBs), polybrominated diphenyl ethers (PBDEs), and polychlorinated dioxins and furans (PCDD/Fs),^8^ tend to accumulate in adipose tissue, making adipose tissue a preferred biological matrix for monitoring chronic POP exposures.^9^

Previous epidemiological studies suggested positive associations between POPs and blood pressure in the general population.^4,10^ However, many of these studies used serum or plasma samples to measure POP exposures. In addition, humans are exposed to multiple chemicals simultaneously at any given time point and cumulatively across their lifetimes. Chemical exposures are highly correlated and potentially have antagonistic or synergistic effects in mixtures.^11−13^ Few studies have examined how mixtures of adipose tissue POPs affect blood pressure, and directions of these associations have mostly been positive but inconsistent. Moreover, the mechanisms underlying the relationship between POP mixtures and blood pressure remain unclear. In vitro studies indicated that POPs in plasma can activate the aryl hydrocarbon receptor (AhR), thereby disrupting AhR-mediated pathways of inflammatory markers,^14−17^ including prostaglandins. POPs act through AhR to modulate prostaglandin pathways, contributing to disruptions in blood pressure regulation.^18−20^ Additionally, POPs can interfere with the renin-angiotensin system and induce oxidative stress, compounding their effects on blood pressure dysregulation.^21,22^ POPs, such as dichlorodiphenyltrichloroethane (DDT), may disrupt the renin-angiotensin-aldosterone system (RAAS) homeostasis by inducing long-term upregulation of renal ion transporters and increasing angiotensin converting enzyme activity. Adipose tissue plays a crucial role in regulating blood pressure through RAAS.^23^ POPs stored in adipose tissue can either exert localized effects within adipose tissue by interfering with RAAS and altering the oxidative microenvironment in adipose tissue^24^ or have systemic effects through the release of lipophilic POPs from the bloodstream by disrupting lipolytic and lipogenic processes, as well as leptin and adiponectin signaling, potentially affecting blood pressure levels.^25,26^

In this study, participants underwent bariatric surgery, an effective treatment for weight loss and cardiometabolic health improvement.^27^ This procedure leads to rapid weight loss and metabolic changes, which result in the release of POPs stored in adipose tissue into the bloodstream.^28−31^ Variability in blood pressure outcomes after bariatric surgery and other weight-loss interventions is well-documented, but the reasons for this heterogeneity remain unclear.^32,33^ One potential explanation lies in the role of cumulative exposure to POPs, which may interact with vascular processes, influencing patients’ blood pressure improvement. Bariatric surgery, a critical intervention for addressing severe obesity and associated cardiometabolic diseases, serves as a model for weight-loss interventions. It not only increases POP levels in the bloodstream but also provides a unique opportunity to evaluate chronic lipophilic POP exposure through adipose tissue samples on blood pressure.

Our primary goal is to estimate the effects of preoperative adipose tissue lipophilic POP mixtures on short-term and long-term blood pressure changes among adolescents with obesity undergoing bariatric surgery. We also utilized adipose tissue metabolomics to better understand the biological mechanism of the health effects of lipophilic POP mixtures on systolic blood pressure (SBP) changes. Metabolomics uniquely provides a direct snapshot of how the human body responds to environmental exposures, offering a holistic view of both physiological and pathophysiological states. A meet-in-the-middle approach supports flexible statistical analysis and reveals biological mechanisms by linking environmental chemical exposures and health outcomes via metabolomic profiles.^34,35^ The knowledge of this study could contribute to the development of targeted strategies to mitigate the adverse health effects associated with lipophilic POP exposures.

## 2 Methods

### 2.1 Study Population

This study leveraged data from the Teen-Longitudinal Assessment of Bariatric Surgery (Teen-LABS) consortium, a prospective, multicenter, observational cohort designed to assess the effectiveness and safety of bariatric surgery in adolescents across the United States. The Teen-LABS study consists of 242 participants with obesity under 19 years of age, who underwent bariatric surgery from March 2007 through February 2012.^36−38^ For this analysis, we included 57 participants with data available on blood pressure measured at baseline, six-month visit, and fifth-year visit as well as adipose tissue metabolomics at baseline. For this substudy of the Teen-LABS cohort, data were available from two medical centers (i.e., Cincinnati Children’s Hospital Medical Center and Texas Children’s Hospital). The University of Southern California Institutional Review Board provided ethical clearance (IRB protocols HS-19-00057). All participants and their guardians provided written informed consent before participating in the study.

### 2.2 Exposure Assessment of Lipophilic POPs

Visceral adipose tissues were frozen in liquid nitrogen and stored at −80 °C until chemical analysis.^39^ We quantified lipophilic POP concentrations with gas chromatography with high-resolution mass spectrometry (GC-HRMS) that allowed quantification using gold-standard isotope dilution methods. C^13^ internal standards were used in each sample. We measured POPs, including *o*,*p*′-dichlorodiphenyldichloroethylene (*o*,*p*′-DDE), *p*,*p*′-DDE, *o*,*p*′-DDT, *p*,*p*′-DDT, hexachlorobenzene (HCB), PCB118, PCB153, PBDE47, PBDE85, and 2,3,7,8-tetrachlorodibenzo-*p*-dioxin (TCDD). *O*,*p*′-DDE, *o*,*p*′-DDT, PBDE85, and 2,3,7,8-TCDD were excluded as their levels were below the limit of detection (LOD) in all samples. The details of the chemical analysis can be found in the supporting infomation.^39^ Values below the LOD were replaced with the LOD divided by the square root of 2.

### 2.3 Blood Pressure Changes after Bariatric Surgery

The primary outcome was percent changes in blood pressure 6 months and five years after bariatric surgery compared to the baseline. The average SBP and diastolic blood pressure (DBP) were taken from ≥2 separate measurements obtained using a Welch Allyn Spot Vital Signs Monitor (4200B, Hillrom, Batesville, Indiana).^38^ Mean arterial pressure (MAP) and pulse pressure were calculated according to MAP = (SBP + 2 × DBP)/3 and pulse pressure = SBP–DBP, respectively. SBP reflects the peak blood pressure during ventricular contraction, while DBP is the lowest pressure measured just before the next contraction. MAP represents average blood pressure during a single cardiac cycle, and pulse pressure provides insights on stiffness of arteries.^40^

### 2.4 Covariates

We identified and selected our covariates *a priori* based on previous literature and listed them in the directed acyclic graph (Figure S1). All analyses were adjusted for: race/ethnicity (non-Hispanic white, others), age at baseline (continuous), patient’s sex (male, female), parents’ annual income (<$25,000, $25,000 to $74,999, ≥ 75,000, unknown), and body mass index (BMI) at baseline (continuous).

### 2.5 Adipose Tissue Metabolome

Adipose tissue samples underwent preparation and analysis in a single batch, including repeated quality control (QC) samples. Initially, up to 30 mg of adipose tissue was treated with 15 μL of 3:1 acetonitrile/water containing ^13^C-labeled internal standards per mg of tissue and then homogenized using a bead beater. The homogenized samples were equilibrated at 4 °C for 30 min and centrifuged at 21,130*g* for 10 min at 4 °C. Two aliquots of the resulting supernatant (30 μL each) were transferred to vials containing either 60 μL of water (for the C18 column) or 60 μL of a 1:1 acetonitrile/water mix (for the hydrophilic interaction liquid chromatography (HILIC) column). These were vortexed briefly and stored in a refrigerated autosampler until analysis.

For untargeted metabolomics analysis, the samples were analyzed by using liquid chromatography with high-resolution mass spectrometry (LC–HRMS). Two separate systems configured for C18 or HILIC analysis were used, featuring a Vanquish Duo Ultra Performance Liquid Chromatography system (Thermo Fisher Scientific, Rockford, IL, USA) coupled to an Exploris120 HRMS system (Thermo Fisher Scientific, Rockford, IL, USA). The column temperatures were maintained at 40 °C for HILIC and 30 °C for C18, while the autosampler was kept at 5 °C. Each sample was analyzed using four analytical configurations: C18 was treated with electrospray ionization (ESI)–, C18 with ESI+, HILIC with ESI–, and HILIC with ESI+. The metabolomic features were extracted using apLCMS with modifications by XMSanalyzer, and the extracted features were defined by the mass-to-charge ratio (*m*/*z*) and retention time as identifiers.

The quality control-based random forest signal correction algorithm from the statTarget R package was applied to remove inter- and intra-batch unwanted variations.^41^ Specifically, features with detection rates below 20% were excluded. Those with a QC sample coefficient of variation greater than 30% were removed, and missing values were imputed using half of the minimum observed values. After QC and preprocessing, we had 21,350 metabolic features from the adipose tissue samples and 3619, 4824, 7615, and 5292 for C18 ESI–, C18 ESI+, HILIC ESI–, and HILIC ESI+, respectively.

We used both our in-house identified metabolite database and MetaboAnalyst for the metabolite annotation.^42^ Metabolite features that were matched to the in-house identified metabolite database were considered as identified metabolites with level-1 confidence.^43^ We had 1973 identified features and 415, 480, 635, and 443 for C18 ESI–, C18 ESI+, HILIC ESI–, and HILIC ESI+, respectively.

### 2.6 Statistical Analysis

We generated descriptive statistics for lipophilic POP concentrations and covariates. The geometric mean (GM) with a 95% confidence interval (CI) was calculated for each POP, along with its distribution. Additionally, Spearman’s correlation coefficients were computed to assess correlations between lipophilic POPs. All lipophilic POPs were log_2_-transformed due to the right-skewedness of the distribution. We used the quantile g-computation to examine the overall effects of the six lipophilic POPs as well as the weights of individual lipophilic POPs on percent changes in blood pressure.^44^ Quantile g-computation provides a way to assess the overall impact of several pollutants on health outcomes, as well as the individual pollutant contributions to health effects. This method involves categorizing exposures into quantiles (e.g., tertiles and quartiles) and then estimating the joint effect of increasing the exposures from one quantile to the next.^44^ We used quartiles of lipophilic POP mixtures to estimate the overall effect on the percent changes in blood pressure at six months and five years after surgery. We also assessed the partial effects, which were the estimates of each POP relative to the overall effect of POP mixtures either in the positive direction or in the negative direction.

We used the meet-in-the-middle approach, which is widely used in omics research, to understand the mechanism underlying lipophilic POP mixtures on blood pressure changes (Figure S2).^34^ Specifically, we first conducted a metabolome-wide association study (MWAS) with adipose tissue POP mixtures via quantile g-computation as predictors, adjusting for sex, race/ethnicity, age at baseline, parents’ annual incomes, and BMI at baseline. The goal was to identify metabolites significantly associated with POP mixtures. We conducted another MWAS using metabolites as predictors and percent changes in selected blood pressure levels as outcomes, adjusting for the same set of covariates, to identify metabolites significantly associated with blood pressure. The selection of blood pressure measures used in MWAS was based on the association between lipophilic POP mixtures and blood pressure changes estimated by quantile g-computation. We summarized significant metabolites with level-1 confidence of associations between lipophilic POP mixtures and selected blood pressure changes.

To account for multiple comparisons, we used both the raw *p*-value at 0.01 and a principal component analysis (PCA)-based methods to determine the α threshold.^45,46^ For each MWAS, PCA was applied to exposures and outcomes separately to calculate the eigenvalues. The effective number of tests (Meff) was then determined by summing all eigenvalues greater than one for both exposures and outcomes according to the Kaiser Guttman rule.^46^ Meff of lipophilic POP mixtures was 2, and Meff of the 21,350 metabolic features was 62. Therefore, the PCA-adjusted *p*-value threshold was 0.00078 for the MWAS with POP mixtures and 0.0008 for the MWAS with the selected blood pressure change.

Sensitivity analyses for overall effects estimation were also conducted. We evaluated the overall effects of the OCP and PCB mixtures separately using quantile g-computation to determine potential differences in chemical class-specific overall effect estimates. The study site was not included as a covariate in the main analysis, but we evaluated it as a potential confounder in the sensitivity analysis.

### 2.7 Pathway Enrichment

Pathway analyses reveal biological processes enriched by significant metabolites.^47^ Based on the results from the MWAS analyses, we used *Mummichog* and gene set enrichment analysis of the MetaboAnalyst to conduct the pathway enrichment analysis. MetaboAnalyst (version 6.0) is used to infer functional activity and metabolic pathways and networks without prior chemical identification.^42^ The algorithm uses *m*/*z* values to match possible metabolites to metabolic features and construct pathways based on tentative identification. Pathways that were independently associated with POP mixtures and blood pressure were identified as overlapped pathways via the meet-in-the-middle approach. Annotated features obtained from pathway enrichment were also identified using the meet-in-the-middle approach after confirmation of *m*/*z* (±10 ppm difference) and retention time (±10 s). Bubble plots were used to visualize the enriched metabolic pathways associated with POP mixtures and blood pressure changes, where each bubble represents the significant hits in the metabolic pathway. Only significant pathways with 3+ significant metabolites were shown in the bubble plot.

All analyses were conducted using R version 4.3.1,^48^ except for the pathway enrichment analysis, which was done using MetaboAnalyst (version 6.0)^42^.

## 3 Results

This study consisted of 57 adolescents with severe obesity undergoing bariatric surgery with adipose tissue POP and blood pressure measured at baseline (Figure S3). The mean age at baseline was 16.91 years (203 months, SD: 18 months), and more than half of the participants were females (72%). Forty-four percent of the participants came from households with annual incomes less than $25,000 (Table 1). The participants’ demographic characteristics were similar to those of the overall cohort (Table S1). All POPs included in this analysis were detected in 95% of participants, and we found higher concentrations for PBDEs (PBDE47) (GM:19.9 ng/g), *p*,*p*′-DDE (GM: 16.1 ng/g), and HCB (GM: 8.34 ng/g, Table 2). Spearman’s correlation coefficients between POP mixtures ranged from −0.2 to 0.89 (Figure S4). The mean and SD of blood pressure percent changes at six months and five years are summarized in Table S2.

**Table 1:** Descriptive Statistics of Participants’ Characteristics in the Teen-LABS Cohort, 2007–2012

characteristic	*N* = 57^a^
**Sex**	
male	16 (28%)
female	41 (72%)
**Age in months at baseline**	203 (18)
**Race**	
Non-Hispanic white	36 (63%)
Others	21 (37%)
**BMI in kg/m**^**2**^**at baseline**	54 (10)
**Parents income category**	
less than $25000	25 (44%)
$25000 to $74999	22 (39%)
$75000 or more	7 (12%)
unknown	3 (5.3%)
**SBP in mmHg at baseline**	127 (15)
**DBP in mmHg at baseline**	76 (11)
**MAP in mmHg at baseline**	93 (11)
**Pulse pressure in mmHg at baseline**	51 (11)

a n (%); mean (SD). Abbreviations: BMI, body mass index; SBP, systolic blood pressure; DBP, diastolic blood pressure; MAP, mean arterial pressure.

**Table 2:** Distribution of Adipose Tissue Lipophilic POP Concentrations (ng/g) in the Teen-LABS Cohort, 2007–2012

lipophilic POPs^a^	detection rate^b^	geometric mean [95% CI]^c^	min	25th percentile	median	75th percentile	max
*p*,*p*′-DDE	100%	16.1 [14.0, 18.6]	0.962	12.4	16.7	23.5	55.2
*p*,*p*′-DDT	100%	0.745 [0.625, 0.888]	0.0953	0.518	0.806	1.27	2.9
HCB	100%	8.34 [8.03, 8.67]	6.26	7.55	8.29	8.94	12.7
PCB118	98.2%	0.683 [0.602, 0.776]	0.0707	0.529	0.69	0.917	2
PCB153	96.5%	0.628 [0.525, 0.752]	0.0707	0.458	0.641	0.946	2.95
PBDE47	98.2%	19.9 [14.5, 27.1]	0.0707	11.4	19.3	31.1	546

a Abbreviations: POP, persistent organic pollutant; *p*,*p*′-DDE, dichlorodiphenyldichloroethylene; *p*,*p*′-DDT, dichlorodiphenyltrichloroethane; HCB, hexachlorobenzene; PCB, polychlorinated biphenyls; PBDE, polybrominated diphenyl ethers.

b The percentage of values above the limit of detection (LOD); values below the LODs were replaced by LOD/√2.

c Unit in ng/g.

### 3.1 Overall Effects of Lipophilic POP Mixtures on Blood Pressure Changes

We evaluated the overall effects of lipophilic POP mixtures on both short-term and long-term changes in blood pressure, measured at six months and five years post bariatric surgery in our study. In the short term, the effects of lipophilic POP mixtures on blood pressure parameters—SBP, DBP, MAP, and pulse pressure—were generally modest. Specifically, the estimated percent changes were 3.52% for SBP (95% CI: −4.24% to 11.3%), −1.17% for DBP (95% CI: −10.9% to 8.53%), 0.85% for MAP (95% CI: −7.28% to 8.98%), and 12.4% for pulse (95% CI: −2.31% to 27.2%) associated with one quartile increase in all lipophilic POPs. In contrast, long-term effects, particularly on SBP, were more pronounced. An increase by one quartile in the lipophilic POP mixtures was associated with a 6.4% increase in SBP (95% CI: 0.4% to 12.4%). Table S3 summarizes individual chemical partial effects, and the significant positive overall effects were primarily driven by PCB153. Long-term changes in DBP, MAP, and pulse over five years were estimated at 4.6% (95% CI: −4.31% to 13.5%), 5.46% (95% CI: −1.48% to 12.4%), and 8.95% (95% CI: −3.36% to 21.3%), respectively (Table 3). In the sensitivity analysis, we evaluated PCB mixtures (PCB118, PCB153) and OCP mixtures (*p*,*p*′*-*DDE, *p*,*p*′*-*DDT, HCB) via quantile g-computation to understand chemical class-based overall effects on blood pressure changes (Table S4). All overall effects of PCBs and OCPs were shown to be positive, but none were statistically significant except for OCP effects on SBP changes five years after bariatric surgery (5.2%, 95% CI: 1%–10.4%). We did not find a significant difference in the overall effects of POP mixtures on blood pressure after adding the study site as an additional covariate (Table S5).

**Table 3:** Adjusted Overall Effects of Lipophilic POP Mixtures and Percent Changes at 6 Months and at Five Years Among Patients in the Teen-LABS Study Using Quantile G-Computation, 2007–2012^a^

BP percent changes	percent changes at 6 months	percent changes at five years
SBP	3.52% (−4.24%, 11.3%)	**6.4% (0.4%, 12.4%)**
DBP	–1.17% (−10.9%, 8.53%)	4.6% (−4.31%, 13.5%)
MAP	0.85% (−7.28%, 8.98%)	5.46% (−1.48%, 12.4%)
pulse pressure	12.4% (−2.31%, 27.2%)	8.95% (−3.36%, 21.3%)

a Note: Coefficients represent overall mixture effects of a simultaneous one quartile increase in all lipophilic POP concentrations from quantile g-computation models. Models were adjusted for race/ethnicity (non-Hispanic white, others), age at baseline (continuous), patient’s sex (male, female), parents’ annual income (<$25,000, $25,000 to $74,999, ≥75,000, unknown), and BMI at baseline (continuous). Abbreviations: POP, persistent organic pollutant; SBP, systolic blood pressure; DBP, diastolic blood pressure; and MAP, mean arterial pressure.

### 3.2 MWAS of Lipophilic POP Mixtures and SBP Changes at Five Years

Table 4 presents the MWAS for lipophilic POP mixtures and the percent changes in SBP five years after bariatric surgery. For lipophilic POP mixtures, we identified 74 and 41 metabolites with a raw *p*-value <0.01 for C18 ESI– and positive ESI+ columns. Similarly, for HILIC, we identified 84 and 63 metabolites with *p* < 0.01 in ESI– and ESI+ modes. After applying PCA-adjusted multiple comparison corrections, eight metabolites remained significant in the C18 columns, and six remained significant in the HILIC columns. Based on significant effect estimates of lipophilic POP mixtures on blood pressure changes, we decided to focus on percent changes in SBP five years postbariatric surgery. We constructed MWAS for fifth year SBP changes and identified 69, 144, 147, and 131 significant metabolites with a *p*-value <0.01 for C18 with ESI–, C18 with ESI+, HILIC with ESI–, and HILIC with ESI+ columns, respectively. After PCA-adjusted corrections, seven metabolites remained statistically significant.

**Table 4:** Number of Significant Metabolites From MWAS of POP Mixtures and SBP Percent Changes with Two Cut off Points in the Teen-LABS Study, 2007–2012

mode^a^	lipophilic POP mixtures^b^		SBP percent changes at five years	
	raw *p*-value at 0.01	PCA-adjusted *p*-value^c^	raw *p*-value at 0.01	PCA-adjusted *p*-value^d^
C18 ESI–	74 (2.04%)	3 (0.08%)	69 (1.9%)	1 (0.03%)
C18 ESI+	41 (0.85%)	5 (0.1%)	144 (2.98%)	5 (0.1%)
HILIC ESI–	84 (1.1%)	3 (0.04%)	147 (1.93%)	1 (0.01%)
HILIC ESI+	63 (1.19%)	3 (0.06%)	131 (2.47%)	0 (0%)

a Note: models were adjusted for race/ethnicity (non-hispanic white, others), age at baseline (continuous), patient’s sex (male, female), parents’ annual income (<$25,000, $25,000 to $74,999, ≥75,000, unknown), and BMI at baseline (continuous).

b The overall effect of the lipophilic POP mixtures was estimated using quantile g-computation.

c PCA-adjusted *p*-values for multiple comparisons indicates the *p*-value cutoff point at 0.00078.

d PCA-adjusted *p*-values for multiple comparisons indicates the *p*-value cutoff point at 0.0008. Abbreviations: MWAS, metabolome-wide analysis study; POP, persistent organic pollutant; SBP, systolic blood pressure; ESI, electrospray ion; HILIC, hydrophilic interaction liquid chromatography; PCA, principal component analysis.

### 3.3 Pathway Enrichment Analysis

**Figure 1:**
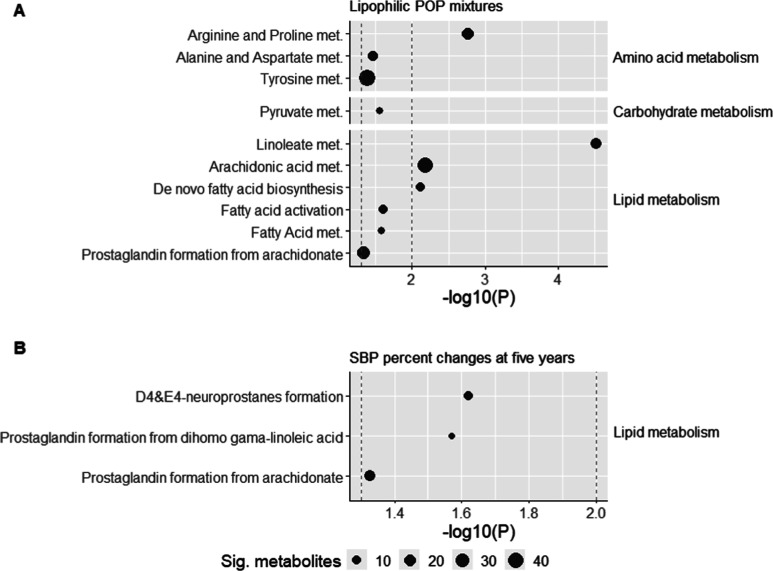
Metabolic pathway enrichment analysis with lipophilic POP mixture and SBP percent changes at five years. Bubble size denotes the total number of significant metabolomic features within each pathway. The log10 (*p*-value) is computed on the *x*-axis. The dashed line denotes a *p*-value threshold of 0.01 and 0.05. Only significant pathways with 3+ significant features are included in this plot (*p*-value <0.05). Pathway enrichment is conducted using MetaboAnalyst (version 6.0).

Because of the limited number of significant features at a raw *p*-value of 0.01 and PCA-based multiple comparisons, we used the *p*-value of 0.05 to include enough features for the following pathway enrichment analyses. Figure 1 illustrates the significant metabolic pathways associated with both lipophilic POP mixtures and SBP changes. Panel A of Figure 1 highlights key biological pathways linked to lipophilic POP mixtures including those involved in amino acid, carbohydrate, and lipid metabolism. Notably, pathways such as arginine and proline metabolism, linoleate metabolism, arachidonic acid metabolism and de novo fatty acid biosynthesis were significantly associated with lipophilic POP mixtures, with a *p*-value of 0.01.

Panel B of Figure 1 highlights pathways significantly associated with changes in SBP at five years. Specifically, pathways related to lipid metabolism, including D4 and E4 neuroprostane formation, prostaglandin formation from dihomo-gamma-linolenic acid, and prostaglandin formation from arachidonic acid, were identified as significant.

### 3.4 Associations of Identified Metabolites with Lipophilic POP Mixtures and SBP Changes

Tables 5 and 6 summarize the associations of identified metabolites with exposure and outcomes. Notably, we identified five amino acids (histidine, acetoacetate, 3-hydroxyanthranilate, asparagine, and indole-3-acetate), two nucleotide derivatives (hypoxanthine and inosine), four lipids (palmitoleate, stearidonic acid, 11-octadecen-9-ynoic acid, and arachidonic acid), and *N*,*N*-dimethylarginine that were negatively associated with adipose tissue lipophilic POP mixtures.

**Table 5:** Identified Metabolites Associated with Lipophilic POP Mixtures in the Teen-LABS Cohort, 2007-2012^a^

						overall effect^b^	partial effects^c^					
group	metabolites	*m*/*z*	RT	column	ψ (95% CI)	*p*-value	*p*,*p*′-DDE	*p*,*p*′-DDT	HCB	PCB118	PCB153	PBDE47
lipid	palmitoleate	253.2173	335.12	C18-	–0.682 (−1.134, −0.229)	0.005	–0.238	0.013	–0.241	0.150	–0.309	–0.056
lipid	[C18.4]-stearidonic acid	275.2017	304.52	C18-	–0.873 (−1.336, −0.409)	0.001	–0.163	–0.075	–0.185	0.019	–0.274	–0.194
lipid	[C18.2]-11-octadecen-9-ynoic acid	277.2173	323.27	C18-	–0.713 (−1.177, −0.249)	0.004	–0.355	0.208	–0.325	–0.038	–0.197	–0.007
lipid	[C20.4]-arachidonic acid	303.2329	340.71	C18-	–0.793 (−1.277, −0.308)	0.002	–0.208	–0.108	–0.233	0.077	–0.146	–0.174
amino acid	histidine	154.0623	18.53	C18-	–0.689 (−1.139, −0.239)	0.004	–0.464	0.309	–0.307	–0.195	0.081	–0.113
amino acid	acetoacetate	101.0242	173.66	HILIC-	–0.779 (−1.26, −0.298)	0.003	–0.319	–0.029	–0.229	–0.048	0.011	–0.164
amino acid	3-hydroxyanthranilate	152.0351	126.77	HILIC-	–0.663 (−1.146, −0.18)	0.010	–0.073	–0.123	–0.234	0.005	–0.162	–0.076
amino acid	asparagine	133.0608	294.41	HILIC+	–0.671 (−1.109, −0.233)	0.004	–0.252	–0.148	–0.191	0.272	–0.281	–0.071
amino acid	indole-3-acetate	176.0706	74.06	HILIC+	–0.729 (−1.234, −0.223)	0.007	–0.150	–0.192	–0.210	0.156	–0.204	–0.130
nucleotide derivatives	hypoxanthine	137.0458	148.33	HILIC+	–0.699 (−1.163, −0.235)	0.005	–0.034	–0.124	–0.186	–0.010	–0.094	–0.252
nucleotide derivatives	inosine	269.0882	183.31	HILIC+	–0.700 (−1.192, −0.208)	0.007	–0.162	–0.270	–0.216	0.004	–0.022	–0.033
others	*N*, *N*-dimethylarginine	203.1503	367.35	HILIC+	–0.614 (−1.054, −0.175)	0.008	–0.217	–0.093	–0.109	0.022	–0.237	0.021

a Note: Models were adjusted for race/ethnicity (non-Hispanic white, others), age at baseline (continuous), patient’s sex (male, female), parents’ annual income (<$25,000, $25,000 to $74,999, ≥75,000, unknown), and BMI at the baseline (continuous).

b Overall effect of the lipophilic POP mixture on adipose tissue metabolome was estimated by quantile g-computation. Estimates are interpreted as the overall mixture effect on the log_2_ intensity of a maternal metabolite for a simultaneous quartile increase in each POPs.

c Partial effects were the estimates of each POP relative to the overall effect of POP mixtures either in the positive direction or the negative direction. Abbreviation: *m*/*z*, mass-to-charge ratio; RT, retention time; POP, persistent organic pollutant; *p*,*p*′-DDE, dichlorodiphenyldichloroethylene; *p*,*p*′-DDT, dichlorodiphenyltrichloroethane; HCB, hexachlorobenzene; PCB, polychlorinated biphenyls; PBDE, polybrominated diphenyl ethers; and HILIC, hydrophilic interaction liquid chromatography.

**Table 6:** Identified Metabolites Associated with SBP Percent Changes at Five Years in the Teen-LABS Cohort, 2007-2012

group^a^	metabolites	*m*/*z*	RT	column	β (95% CI)	*p*-value
amino acid	hippuric acid	178.0509	23.40	C18-	–3.88% (−7.24%, −0.524%)	0.029
amino acid	*N*-acetylglutamate	188.0563	303.47	HILIC-	4.07% (1.13%, 7.01%)	0.010
amino acid	indole-3-acetate	176.0706	74.06	HILIC+	–3.49% (−6.51%, −0.48%)	0.029
others	choline	139.0765	43.83	C18-	–3.49% (−6.62%, −0.37%)	0.034

a Note: models were adjusted race/ethnicity (non-Hispanic white, others), age at baseline (continuous), patient’s sex (male, female), parents’ annual income (<$25,000, $25,000 to $74,999, ≥75,000, unknown), and BMI at the baseline (continuous). Abbreviation: SBP, systolic blood pressure; *m*/*z*, mass-to-charge ratio; RT, retention time; HILIC, hydrophilic interaction liquid chromatography.

We identified three amino acids (hippuric acid, *N*-acetylglutamate, and indole-3-acetate) associated with SBP percent changes five years postbariatric surgery. Among these, *N*-acetylglutamate showed a positive association, while hippuric acid, indole-3-acetate, and choline were negatively associated with SBP changes.

### 3.5 Meet-In-The-Middle Approach

**Figure 2:**
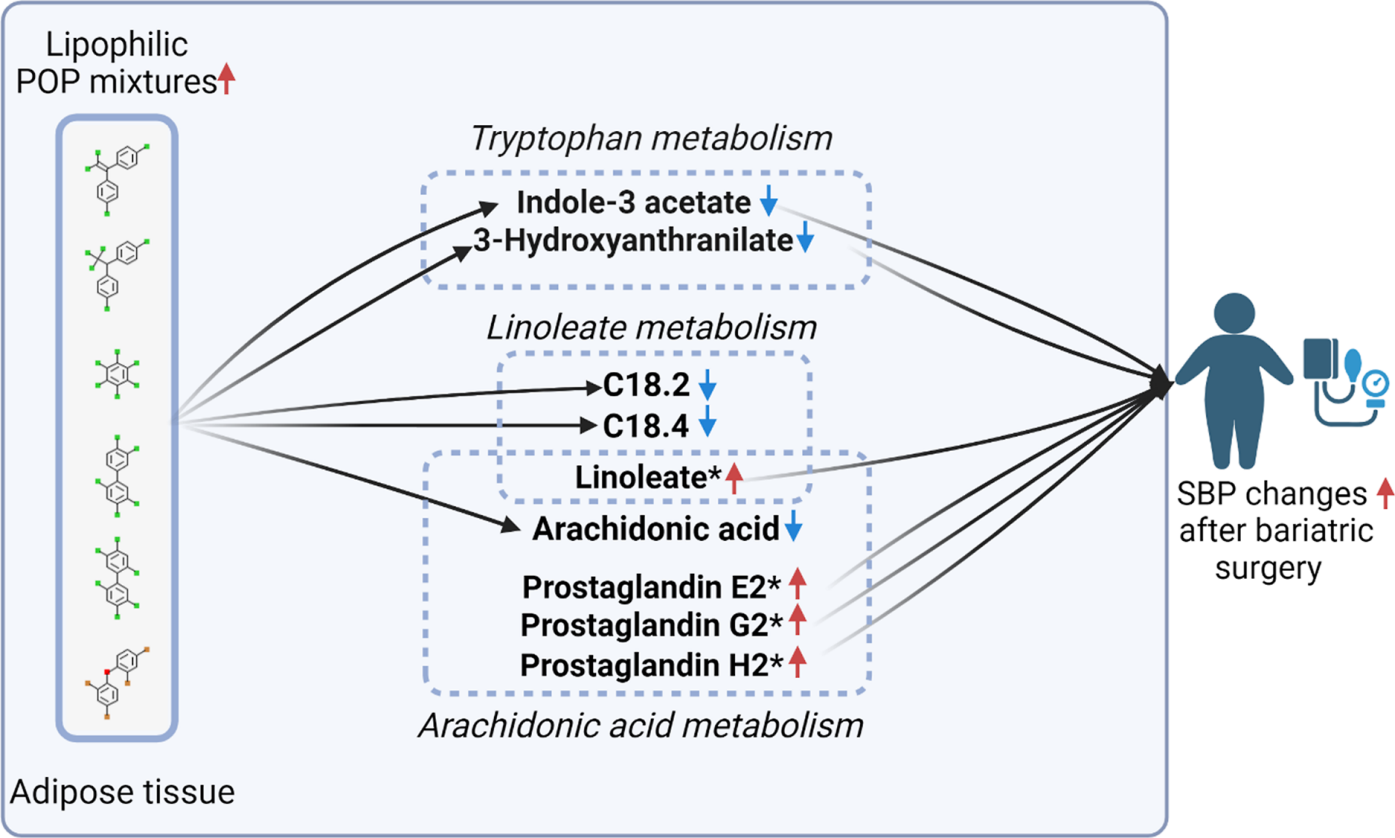
Mechanistic meet-in-the-middle plot of the associations between lipophilic POP mixtures and SBP changes five years following bariatric surgery. Asterisk indicated metabolites that were annotated metabolites from MetaboAnalyst (version 6.0). All the other metabolites were identified with level 1 confidence. Blue arrows indicated negative associations, and red arrows indicated positive associations.

We compared both identified metabolites and annotated metabolites significantly associated with adipose tissue lipophilic POP mixtures and SBP changes. Annotated metabolites were putatively identified through pathway enrichment analysis using MetaboAnalyst, and significant annotated features are presented in Tables S6 and S7. Our analysis revealed one identified metabolite, indole-3-acetate, that was significantly associated with both POP mixtures and SBP changes. Indole-3-acetate was negatively associated with both lipophilic POP mixtures and SBP changes. With one simultaneous quartile increase in POP mixtures, the log_2_ intensity of indole-3-acetate decreased by 0.729 (95% CI: −1.234, −0.223). With a 2-fold increase in the intensity of indole-3-acetate, SBP decreased 3.49% five years after bariatric surgery (95% CI: −6.51%, −0.48%). Additionally, we identified key metabolites involved in overlapping biological pathways, including linoleic acid metabolites (C18.2 and C18.4), arachidonic acid from the linoleate metabolism pathway, and prostaglandins from the arachidonic acid metabolism pathway. Significant metabolites within overlapping pathways are presented in Figure 2.

## 4 Discussion

In this study, we examined the overall effects of lipophilic POP mixtures on short- and long-term blood pressure changes in a cohort of adolescents undergoing bariatric surgery. We used high-resolution adipose tissue metabolomics to understand the underlying mechanism of lipophilic POP mixtures in adipose tissue on SBP changes. We observed a significant, positive association between lipophilic POP mixtures and SBP changes five years after bariatric surgery. Within this association, we identified a negative relationship between lipophilic POP mixtures and the metabolite indole-3-acetate, which, in turn, was negatively associated with long-term SBP changes. Our analysis revealed that prostaglandin formation was a key biological pathway through which lipophilic POP mixtures influenced SBP changes five years after bariatric surgery. We also identified key metabolites (e.g., C18.2, C18.4, arachidonic acid, linoleate, prostaglandin E2, prostaglandin G2, and prostaglandin H2) in tryptophan, linoleate, and arachidonic acid metabolism that were either associated with POP mixtures or SBP changes. We found no statistically significant associations of lipophilic POP mixtures with any blood pressure changes six months after bariatric surgery or with DBP, MAP, and pulse pressure changes five years after bariatric surgery.

In general, the POP concentrations measured in adipose tissue were lower in the Teen-LABS compared to POP concentrations measured in adipose tissue among the adult population in the United States, Europe, and China.^49−53^ Very few studies have reported adipose tissue POP concentrations among adolescent populations. However, compared to another adolescent population undergoing bariatric surgery, the *p*,*p*′*-*DDE, HCB, PCB153, and PBDE47 concentrations in visceral adipose tissue were consistently higher in the Teen-LABS cohort.^54^

Previous studies on the blood pressure effects of individual POPs and POP mixtures measured in plasma and adipose tissue have inconsistent findings. PCBs and *p,p'*-DDE were found positively associated with high blood pressure or hypertension risk, while some reported negative associations between hexachlorocyclohexane (HCH) congeners and hypertension.^10,51,55−57^ POPs in adipose tissue were positively associated with hypertension risk among adults with BMI over 26.3 kg/m^2^.^56^ For PBDEs, PBDE28 and PBDE71 were positively associated with DBP.^51^ Among adolescent populations, a recent study found that beta-HCH and *p*,*p*′*-*DDE were positively associated with SBP among teenage girls.^57^ Although no studies have reported associations between POP mixtures in adipose tissue and blood pressure, one cross-sectional study examined the association between POP mixtures in adipose tissue and metabolic syndrome and found that higher levels of POP mixtures in adipose tissue were statistically significantly associated with increased odds of metabolic syndrome, with gamma hexachlorocyclohexane (gamma-HCH), *o*,*p*′*-*DDT, and HCB contributing the most to this effect. HCB and gamma-HCH exposures were consistently associated with higher blood pressure.^52^ This is consistent with our study in which we found higher HCB and DDE concentrations were associated with higher percent changes in blood pressure. We also found the non dioxin-like PCB153 to be positively associated with blood pressure changes.

Prostaglandin formation from arachidonate was the significant biological pathway that was associated with both POP mixtures and SBP changes after bariatric surgery. Prostaglandins are lipid autacoids derived from arachidonic acid and formed via cyclooxygenase (COX) enzymes. Along with arachidonic acid, prostaglandin plays key roles in sustaining homeostatic functions and mediating inflammatory responses.^58^ COX-2, also known as prostaglandin G/H synthase 2, is typically induced during inflammation and is responsible for producing proinflammatory prostaglandins such as prostaglandin E2. However, COX-2-derived prostaglandins can have both proinflammatory and anti-inflammatory effects on immune cells. DDE and other lipophilic POPs in adipose tissue can modulate COX-2 synthesis and contribute to the chronic inflammation.^59−61^ Unfortunately, we are unaware of studies investigating the prostaglandin formation mechanism in response to mixture-based POP exposures. Epidemiologic evidence also showed that POPs accumulated in adipose tissue might relate to the adipose tissue macrophage infiltration and inflammation.^62^ At the same time, prostaglandin has long been proved to be involved in blood pressure regulation.^63,64^ Noticeably, we also found that lipid-related pathways enriched in the lipophilic POP mixtures with the lowest *p*-values were linoleate and arachidonic acid metabolism. Linoleic acid is an omega-6 fatty acid, which is a substrate for prostaglandin formation.^65^ This evidence suggests that prostaglandin synthesis via COX enzymes may play an important role in the mechanism underlying POP mixture effects on blood pressure regulation.

Adipose tissue POP mixtures were associated with substrate metabolism pathways including amino acid, carbohydrate, and lipid metabolism. This was consistent with a previous study on POP mixtures in adipose tissue,^54^ in which adipose tissue POPs were correlated with amino acid metabolism, lipid and fatty acid metabolism, and carbohydrate metabolism. In the case of amino acid metabolism, the tyrosine metabolite 3,4-dihydroxymandelate, and the tryptophan metabolites indole-3-acetate and 3-hydroxyanthranilate were significant metabolites involved in associations with both POP mixtures and SBP changes. Indeed, tryptophan and its metabolites have a basis in blood pressure regulation, as evidenced by hypertension caused in rats fed tryptophan and by the elevation of several tryptophan metabolites in hypertensive rats but not in control rats.^66−68^ Importantly, tryptophan metabolites, including indole-3-acetic acid, and our POP mixture component PCB118 activate the AhR,^69^ and knockout of the aryl hydrocarbon elevates blood pressure in mice.^70^ This evidence raises the possibility that tryptophan metabolite levels influenced by POP mixtures in adipose tissue may reflect systemic changes in AhR signaling that ultimately attenuate postsurgery improvements in blood pressure.

The major strength of this study lies in its unique design and sample characteristics, which enabled the investigation of tissue-specific exposures and their association with tissue-specific metabolic profiles in relation to blood pressure changes. Lipophilic POPs are rapidly mobilized from adipose tissue and released into the bloodstream following a bariatric surgery. This surgery amplified the typically subtle effects of POP mixtures on health outcomes observed in the general population. The findings from this study provide valuable insights into how lipophilic POP mixtures influence changes in blood pressure.

However, this study has several limitations. First, the small sample size reduced our statistical power. This potentially hinders the detection of significant associations of DBP, MAP, and pulse pressure changes after surgery. Although previous studies have reported sex-specific associations between POPs and blood pressure, we lacked sufficient statistical power to perform sex-stratified analyses.^57^ Second, we assumed linear monotonic relationships between lipophilic POP mixtures and blood pressure changes due to the limited sample size and constraints of the metabolomics integration design. Third, we were limited in our ability to control for some time-varying confounding factors. BMI was adjusted as a baseline variable rather than time-varying, which might introduce residual confounding. Dietary information was only available for a subset of participants; thus, we were unable to control for it in analyses; however, we assumed this would have a minimal impact on the overall effects estimation of POP mixtures on blood pressure changes (Figure S1B). Patients’ postsurgery dietary patterns were unlikely to be associated with POP exposures stored in adipose tissue at baseline, as patients need to adhere to specific dietary guidelines after major interventions like bariatric surgery.^71^ Nonetheless, further research is needed to explore how time-varying dietary patterns might influence the association between lipophilic POP mixtures and blood pressure changes as well as the underlying mechanisms involved.

In summary, lipophilic POP mixtures were positively associated with SBP changes measured five years after bariatric surgery. Prostaglandin formation from arachidonic acid via COX enzymes and amino acid metabolites, including those of tryptophan metabolism, may play an important role in this association.

These findings provided valuable insights that could inform public health policies aimed at mitigating the adverse health effects of environmental chemical exposure on individuals with obesity, particularly those pursuing weight-loss interventions.

## Supporting Information Available

Chemical analysis of lipophilic POPs in visceral adipose tissue. Internal standards of adipose tissue metabolome. Table S1: Descriptive statistics of the analytic dataset versus the whole cohort in the Teen-LABS study, 2007–2012. Table S2: Summary statistics of blood pressure percent changes at 6 months and 5 years following bariatric surgery in the Teen-LABS study, 2007–2012. Table S3: Adjusted overall effects of lipophilic POPs and percent changes at 5 years after bariatric surgery among patients in the Teen-LABS study. Table S4: Adjusted overall effects of OCPs and PCBs with percent changes at 6 months and 5 years among patients in the Teen-LABS study. Table S5: Adjusted overall effects of lipophilic POP mixtures and percent changes at 6 months and 5 years among patients in the Teen-LABS study. Table S6: Annotated metabolites significantly associated with lipophilic POP mixtures in the Teen-LABS cohort, 2007–2012. Table S7: Annotated metabolites significantly associated with SBP percent changes at 5 years after bariatric surgery in the Teen-LABS cohort, 2007–2012. Figure S1: Directed acyclic graph: (A) The association between lipophilic POP mixtures and adipose tissue metabolomics; (B) The association between adipose tissue metabolomics and blood pressure changes after bariatric surgery. The green line indicates the causal path, and the red lines indicate the biasing paths. AT, Adipose tissue; BMI, Body mass index. Figure S2: Analytical workflow of AT metabolomics. Figure S3: Population flowchart of the Teen-LABS cohort. Figure S4: Spearman’s correlation between adipose tissue lipophilic POP mixtures (PDF)
