# Supplementary material for: Metabolic Signatures in Adipose Tissue Linking Lipophilic Persistent Organic Pollutant Mixtures to Blood Pressure Five Years After Bariatric Surgery Among Adolescents
Source: Environ Sci Technol. 2025 Feb 25;59(9):4364–75. doi: 10.1021/acs.est.4c13902 (PMC11912326; doi:10.1021/acs.est.4c13902)
Supplement: Supplementary file 1 — es4c13902_si_001.pdf [file es4c13902_si_001.pdf]

Supporting Information

Metabolic signatures in Adipose Tissue linking Lipophilic Persistent Organic Pollutant Mixtures to Blood Pressure Five Years after Bariatric Surgery among Adolescents

Shudi Pan[1], Zhenjiang Li[1*], Douglas I. Walker[2], Brittney O. Baumert[1], Hongxu Wang[1], Jesse A. Goodrich[1], Sarah Rock[1], Thomas H. Inge[3], Todd M. Jenkins[4], Stephanie Sisley[5], Scott M. Bartell[6], Stavra Xanthakos[7], Xiangping Lin[8], Brooklynn McNeil[9], Anna R. Robuck[10], Catherine E. Mullins[2], Michele A. La Merill[11], Erika Garcia[1], Max T. Aung[1], Sandrah P. Eckel[1], Rob McConnell[1], David V. Conti[1], Justin R. Ryder[3], Lida Chatzi[1]

Affiliations:

1. Department of Population and Public Health Sciences, Keck School of Medicine, University of Southern California, Los Angeles, CA 90032, USA
2. Gangarosa Department of Environmental Health, Emory University, Atlanta, GA 30322, USA
3. Department of Surgery, Northwestern University Feinberg School of Medicine and Ann & Robert H. Lurie Children's Hospital of Chicago, Chicago, IL 60611, USA
4. Division of Biostatistics & Epidemiology, Cincinnati Children's Hospital Medical Center, Department of Pediatrics, University of Cincinnati College of Medicine, Cincinnati, OH 45229, USA
5. USDA/ARS Children's Nutrition Research Center, Department of Pediatrics, Baylor College of Medicine, Houston, TX 77030, USA
6. Department of Environmental and Occupational Health, Department of Epidemiology and Biostatistics, and Department of Statistics, University of California, Irvine, CA 92697, USA
7. Division of Gastroenterology, Hepatology, Nutrition, Cincinnati Children's Hospital Medical Center, Department of Pediatrics, University of Cincinnati College of Medicine, Cincinnati, OH 45229, USA
8. Department of Genetics, Stanford University School of Medicine, Stanford, CA 94305, USA
9. Irving Institute for Clinical and Translational Research, Columbia University, New York, NY 10027, USA
10. Department of Environmental Medicine and Public Health, Icahn School of Medicine at Mount Sinai, New York, NY 10029, USA
11. Department of Environmental Toxicology, University of California, Davis, CA 95616, USA

Number of pages: 13

Number of figures: 4

Number of tables: 7

Chemical analysis of lipophilic POPs in visceral adipose tissue

Briefly, approximately 0.15g of tissue was weighed, placed in plastic vial containing 10 glass beads and treated with 3.5mL of LC-MS grade water. Each sample was spiked with 12.5µL of 2-proponal solution containing 100 ng/mL 13C-labelled internal standards, and then homogenized using six cycles of 15-sec on/15-sec off in a BioSpec Mini-Beadbeater-24 maintained at 4ºC. The homogenate was sonicated for 10 min in a sonicating bath, and then transferred to a 50 mL conical tube containing 10mL acetonitrile, 4000mg MgSO4 and 1000mg NaCl, and vortexed briefly immediately after. The original homogenate tube was rinsed with another 3.5 mL of water, vortexed and added to 50mL vial with extraction salts. The mixture was vortexed for 5 min, and then centrifuged for 5 mins at 3,900 rpm. 8 mL of the resulting supernatant was transferred to a clean, 15mL tube, and stored overnight at -20ºC to precipitate remaining lipids. Following overnight storage, the tubes were centrifuged for 5 min at 4ºC and a 4.5mL aliquot of supernatant was then transferred to a 15mL tube containing 300 mg primary and secondary amine exchange material (PSA), 300 mg C18 and 900 mg MgSO4. The extract and sorbents were vortex-mixed for 5 min and centrifuged for 5 min. Following cleanup, 2.5ml of supernatant was evaporated and solvent exchanged to toluene under a gentle stream of N2 to 150µL. The extract was then transferred to a GC vial containing a low volume insert and stored at -20C until analysis. Tissue extracts were analyzed using a Thermo Scientific 1310 gas chromatograph connected to a Q Exactive GC Orbitrap GC-MS/MS ultra-high-resolution mass spectrometer and Triplus RSH autosampler. A 2 µL aliquot of extract was injected into an inlet maintained at 250ºC in pulsed split-less mode. The analytes were separated on an Agilent DB-5MSUI capillary column (30m length × 0.25mm inner diameter × 0.25µm film thickness) using high purity helium (99.999% purity) as the carrier gas at a constant flow rate of 1 mL/min. The oven temperature program consisted of an initial temperature of 100ºC for 1 min, increased to 180ºC at 25ºC/min; followed by a temperature ramp to 215ºC at 5ºC/min, and finally increased to 300ºC at 25ºC/min and held for 10 min, resulting in a total run time of 26.6 min.

Mass spectral data were obtained using an electron ionization source, operated at a source temperature of 250°C, electron impact energy of -70 eV and transfer line temperature maintained at 280°C. The UHRMS

was operated in full-scan mode over mass-to-charge (m/z) range 85-850 and 60,000 resolution. DDE concentrations were determined using an isotope dilution approach in combination with a five-point calibration curve. Only peaks within the analyte retention time +/- 0.2 minutes were considered for quantification. The method detection limit (MDL) was determined based on the average concentration detected in process blanks or the lowest curve point with a signal-to-noise ratio exceeding 10; the higher value was selected for the MDL. The limit of detection (LOD) for lipophilic POPs was established as 0.10 ng/g, except for HCB (LOD = 12.07 ng/g) and PBDE85 (LOD = 1 ng/g). The LOD for HCB was determined using blank controls, which may have resulted in an overestimation. As a result, values below the LOD were also considered in this analysis.

Internal standards of adipose tissue metabolome

**Standard**	**Short Name**	**CAS**	**Vendor**	**Part Number**
Cortisol (2, 3, 4-13C3)	Cortisol	19996649-39-8	Cambridge Isotope	CLM-10371-C
NNAL (1, 2’, 3’, 4’, 5’, 6’-13C6)	NNAL	NA	Cambridge Isotope	CLM-4556-1.2
Caffeine (trimethyl-13C3)	Caffeine	78072-66-9	Cambridge Isotope	CLM-514-1
L-Methionine (13C5)	Methionine	202326-57-6	Cambridge Isotope	CLM-893-H-0.05
DL-Cotinine (2’, 3’, 4’-13C3)	Cotinine	NA	Cambridge Isotope	CLM-9692-1.2
L-Tyrosine (13C9, 15N)	Tyrosine	202407-26-9	Cambridge Isotope	CNLM-439-H-0.1
L-Glutamic Acid (13C5, 15N)	Glutamic Acid	NA	Cambridge Isotope	CNLM-554-H-0.25
D-Glucose (U-13C6)	Glucose	110187-42-3	Cambridge Isotope	CLM-1396-1
L-Cystine (3, 3’-13C)	Cystine	2483736-13-4	Cambridge Isotope	CLM-520-0.25
DL-Nicotine (3’, 4’, 5’-13C3)	Nicotine	202114-61-2	Cambridge Isotope	CLM-3914-0.1

L-Arginine:HCL (13C6)	Arginine	201740-91-2	Cambridge Isotope	CLM-2265-H-0.25
Perfluoro-n-[13C8]octanoic acid	PFOA	1350614-84-4	Wellington Laboratories	M8PFOA
Sodium perfluoro-[13C8]octanesulfonate	PFOS	2522762-16-7	Wellington Laboratories	M8PFOS
Perfluoro-n-[13C9]nonanoic acid	PFNA	2283397-80-6	Wellington Laboratories	M9PFNA
Perfluoro-n-[1,2,3,4,6-13C5]hexanoic acid	PFHxA	2328024-54-8	Wellington Laboratories	M5PFHxA
Perfluoro-n-[1,2,3,4,5,6-13C6]decanoic acid	PFDeA	2328024-56-0	Wellington Laboratories	M6PFDA
Perfluoro-n-[1,2,3,4,5,6,7-13C7]undecanoic acid	PFUDeA	NA	Wellington Laboratories	M7PFUdA
Sodium perfluoro-1-[1,2,3-13C3]hexanesulfonate	PFHxS	2708218-86-2	Wellington Laboratories	M3PFHxS
Perfluoro-n-[2,3,4-13C3]butanoic acid	PFBA	2483735-33-5	Wellington Laboratories	M3PFBA

Table S1: The descriptive statistics of the analytic dataset vs the whole cohort in the Teen-LABS study, 2007-2012

**Characteristic**	Analytic subset<br>**N = 57**[1]	Overall<br>**N = 220**[1]
**Sex**		
male	16 (28%)	54 (25%)
female	41 (72%)	166 (75%)
**Age in months at baseline**	203 (18)	205 (18)
**Study site**[2]		
A	22 (39%)	59 (27%)
B	35 (61%)	90 (41%)
**Race**		
others	21 (37%)	64 (29%)
non-Hispanic white	36 (63%)	156 (71%)
**BMI in kg/m^2 at baseline**	54 (10)	53 (9)
unknown		28
**Parents income category**		
less than $25000	25 (44%)	68 (31%)
$25000 to $74999	22 (39%)	71 (32%)
$75000 or more	7 (12%)	43 (20%)
unknown	3 (5.3%)	38 (17%)

[1]n (%); Mean (SD)

[2]Locations were denoted as A and B to maintain confidentiality in accordance with Institutional Review Board requirements.

Table S2: The summary statistics of blood pressure percent changes at 6 months and five years following bariatric surgery in the Teen-LABS study, 2007-2012

**Blood pressure**	**6 months**[1]	**Five years**[1]
SBP	-0.04 (0.14)	-0.02 (0.12)
DBP	-0.06 (0.14)	0.01 (0.18)
MAP	-0.05 (0.12)	-0.01 (0.14)
Pulse	0.03 (0.29)	-0.05 (0.25)

[1]Mean (SD)

Abbreviations: SBP, Systolic blood pressure; DBP, Diastolic blood pressure; MAP, Mean arterial pressure.

Table S3: Adjusted overall effects of lipophilic POPs and percent changes at five years after bariatric surgery among patients in the Teen-LABS study using quantile g computation, 2007-2012

	**SBP percent changes at five years**	
ψ[1]	6.4% (0.4%,12.4%)	
Direction	Positive[2]	Negative[3]
Scaled effect	9.26%	-2.85%
Partial effects[4]		
p,p'-DDE[4]	2.32%	-
p,p'-DDT	-	-0.53%
HCB weight	2.67%	-
PCB118 weight	-	-1.32%
PCB153 weight	4.27%	-
PBDE47 weight	-	-1.00%

Note: Models were adjusted for race (non-Hispanic white, others), age at baseline (continuous), patient's sex (male, female), parents' annual income (< \$25,000, \$25,000 to \$75,000, ≥ 75,000, unknown) and BMI at baseline (continuous).

[1]Difference (95% confidence interval) in blood pressure outcome % mmHg per quartile increases in all lipophilic POPs.

[2]Difference in blood pressure outcome % mmHg per quartile increases in all lipophilic POPs with estimated effects in the positive direction.
[3]Difference in blood pressure outcome % mmHg per quartile increases in all lipophilic POPs with estimated effects in the negative direction.
[4]Partial effects of each lipophilic POPs relative to the overall mixture effect.

Table S4: Adjusted overall effects of OCPs and PCBs with percent changes at 6 months and five years among patients in the Teen-LABS study using quantile g-computation**,** 2007-2012[1]

BP	Chemical class	percent changes at 6 months	percent changes at five years
SBP	OCP	0.04 (-0.03,0.111)	**0.052 (0.001,0.104)**
SBP	PCB	0.027 (-0.021,0.075)	0.02 (-0.019,0.06)
DBP	OCP	0.002 (-0.078,0.082)	0.023 (-0.057,0.103)
DBP	PCB	0.005 (-0.056,0.066)	0.026 (-0.035,0.088)
MAP	OCP	0.02 (-0.05,0.089)	0.037 (-0.024,0.098)
MAP	PCB	0.014 (-0.037,0.064)	0.023 (-0.023,0.07)
Pulse	OCP	0.096 (-0.038,0.231)	0.092 (-0.016,0.2)
Pulse	PCB	0.084 (-0.011,0.178)	0.011 (-0.075,0.098)

[1]Note: Coefficients represent mixture joint effects of a simultaneous 1 quantile increase in OCP and PCB mixtures from from quantile g-computation models. Models were adjusted for race (non-Hispanic white, others), age at baseline (continuous), patient's sex (male, female), parents' annual income (< $25,000, $25,000 to $75,000, ≥ 75,000, unknown) and BMI at baseline (continuous).

Abbreviations: POP, Persistent Organic Pollutant; OCP, Organochlorine pesticides; PCB, Polychlorinated biphenyls; SBP, Systolic blood pressure; DBP, Diastolic blood pressure; MAP, Mean arterial pressure.

Table S5: Adjusted overall effects of lipophilic POP mixtures and percent changes at 6 months and five years among patients in the Teen-LABS study using quantile g-computation after adding study sites as a covariate, 2007-2012[1]

BP percent changes	percent changes at 6 months	percent changes at five years
SBP	2.75% (-3.87%,9.37%)	**6.32% (0.24%,12.4%)**
DBP	-2.03% (-10.68%,6.62%)	4.4% (-4.56%,13.36%)
MAP	0.04% (-6.88%,6.95%)	5.31% (-1.67%,12.29%)
Pulse	11.75% (-2.78%,26.27%)	9.05% (-3.45%,21.54%)

[1]Note: Coefficients represent overall mixture effects of a simultaneous one quartile increase in all lipophilic POP exposures from quantile g-computation models. Models were adjusted for race (non-Hispanic white, others), age at baseline (continuous), patient's sex (male, female), parents' annual income (< \$25,000, \$25,000 to \$75,000, ≥ 75,000, unknown), BMI at baseline (continuous) and study site (binary).

Abbreviations: POP, Persistent Organic Pollutant; SBP, Systolic blood pressure; DBP, Diastolic blood pressure; MAP, Mean arterial pressure.

Table S6: Annotated metabolites associated with lipophilic POP mixtures in the Teen-LABS cohort, 2007-2012

Group	Metabolites[2]	m/z	RT	Column	Psi[3]	p-value
Amino acid	Proline	135.03	126.93	HILIC-	-0.73	0.00271
Amino acid	Citrulline	130.10	383.79	HILIC+	-0.60	0.00978
Amino acid	2-Phenylacetamide	118.07	31.04	C18+	-0.94	0.00017
Amino acid	Norsalsolinol	146.06	247.11	HILIC-	-0.67	0.00550
Amino acid	L-Tyrosine	164.07	417.61	HILIC+	0.77	0.00332
Amino acid	3,4-Dihydroxymandelic acid	165.02	29.10	C18-	-0.73	0.00178
Amino acid	Norepinephrine sulfate	248.02	142.37	HILIC-	-0.65	0.00748
Lipid	Leukotriene F4 cytosol	567.27	386.72	HILIC+	-0.64	0.00845
Lipid	Prostaglandin B1	336.23	238.25	C18-	-0.77	0.00331
Lipid	Prostaglandin E1	353.23	210.30	C18-	-0.81	0.00136
Lipid	Eicosapentaenoic acid	302.22	268.60	C18-	-0.74	0.00302
Lipid	Myristic acid	287.22	57.59	HILIC-	0.67	0.00495
Lipid	(E)-4-oxonon-2-enal	155.11	401.76	C18+	-0.75	0.00268
Lipid	Prostaglandin A2	335.22	68.44	HILIC+	-0.67	0.00762

[1]Note: Models were adjusted race/ethnicity (non-Hispanic white, others), age at baseline (continuous), patient's sex (male, female), parents' annual income (< $25,000, $25,000 to $75,000, ≥ 75,000, unknown) and BMI at baseline (continuous).

[2]Annotated metabolites in significant pathways were putatively matched based on Metaboanalyst database via the m/z and experimental m/z. RT was reported in seconds.

[3]Overall effect of the lipophilic POP mixture on adipose tissue metabolome was estimated by quantile g-computation. Estimates are interpreted as the overall mixture effect on the $\log_2$ intensity of a maternal metabolite for a simultaneous quartile increase in each POPs.

Table S7: Annotated metabolites associated with SBP percent changes at five years after bariatric surgery in the Teen-LABS cohort, 2007-2012

Group	Metabolites[2]	m/z	RT	Column	Beta	p-value
Lipid	Dehydroascorbic acid	210.98	17.48	HILIC-	0.04	0.039
Lipid	Linoleic acid	325.24	348.93	C18-	0.03	0.046
Lipid	Prostaglandin G2	183.10	57.09	HILIC-	-0.03	0.048
Lipid	Prostaglandin E2	372.19	39.87	HILIC-	0.04	0.012
Lipid	Prostaglandin H2	397.22	224.03	C18-	0.05	0.001
Lipid	11-dihydro-leukotriene B4	352.22	428.38	C18-	0.05	0.014
Lipid	Prostaglandin F2a	369.23	215.79	C18-	0.05	0.014

[1]Note: Models were adjusted race/ethnicity (non-Hispanic white, others), age at baseline (continuous), patient's sex (male, female), parents' annual income (< \$25,000, \$25,000 to \$75,000, ≥ 75,000, unknown) and BMI at baseline (continuous).

[2]Annotated metabolites in significant pathways were putatively matched based on Metaboanalyst database via the m/z and experimental m/z. RT was reported in seconds.

Abbreviations: SBP, Systolic blood pressure; DBP, Diastolic blood pressure; MAP, Mean arterial pressure.

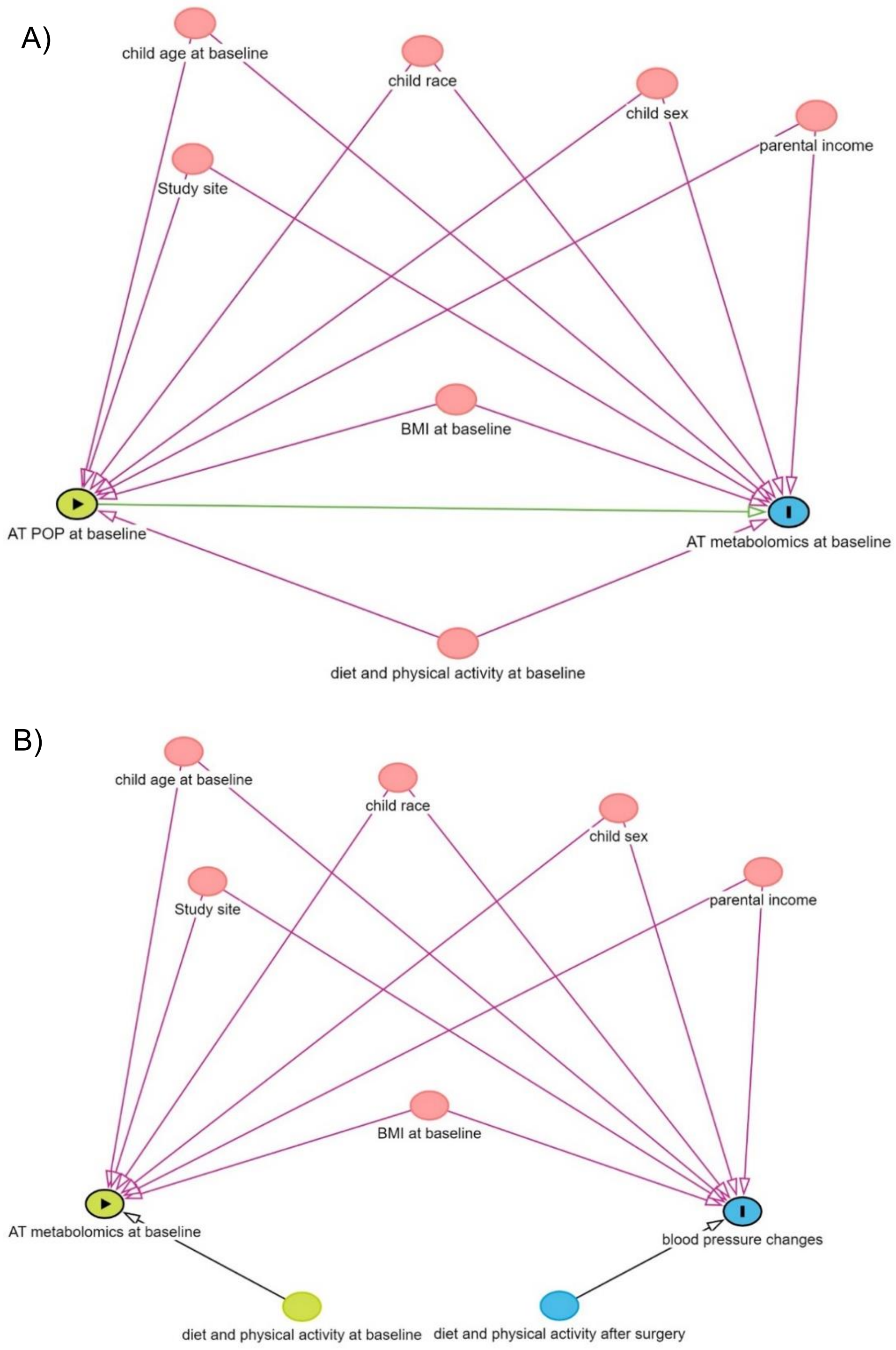

Figure S1: Directed acyclic graph: A) The association between lipophilic POP mixtures and adipose tissue metabolomics; B) The association between adipose tissue metabolomics and blood pressure changes after bariatric surgery. The green line indicates the causal path and the red lines indicate the biasing paths.
AT, Adipose tissue; BMI, Body mass index

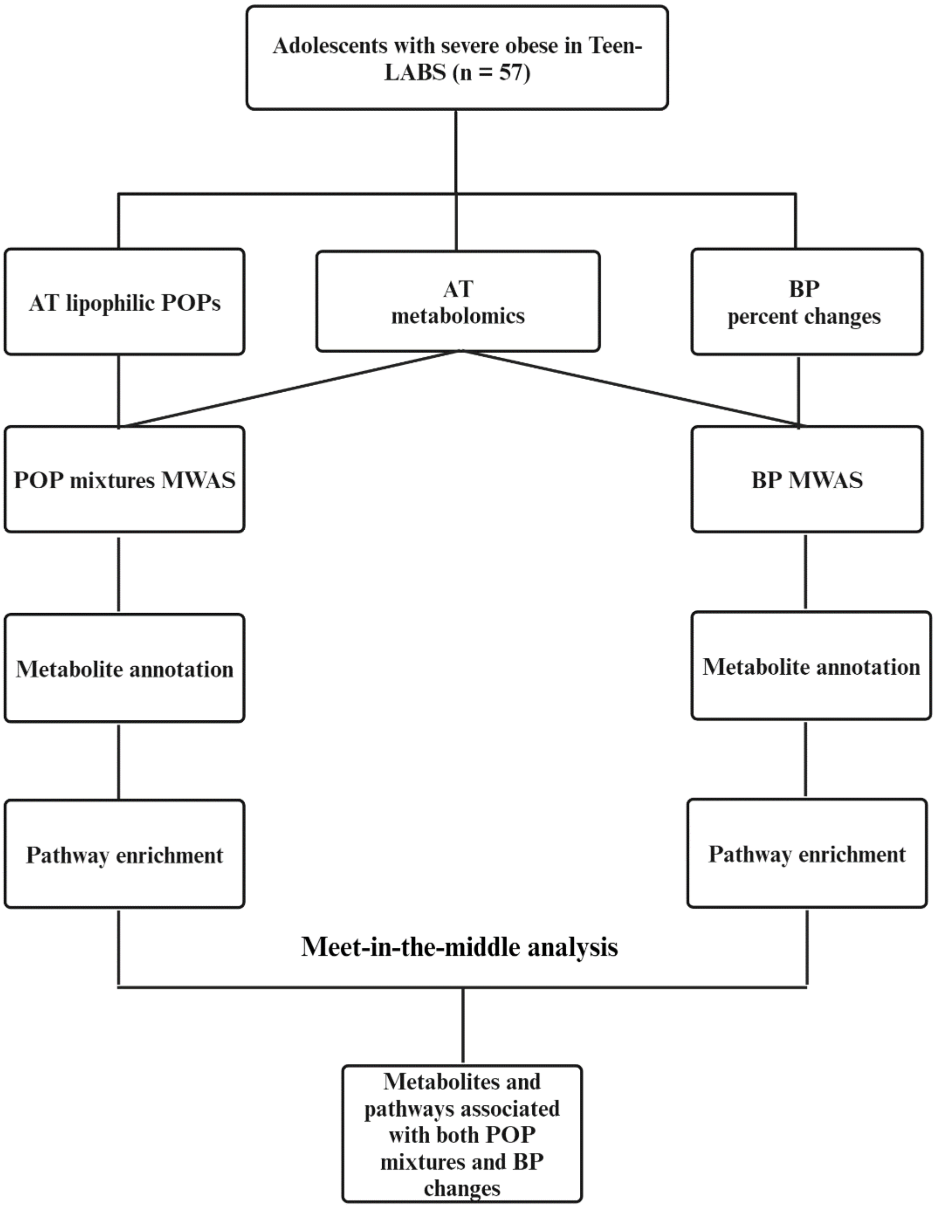

Figure S2: Analytical workflow of AT metabolomics

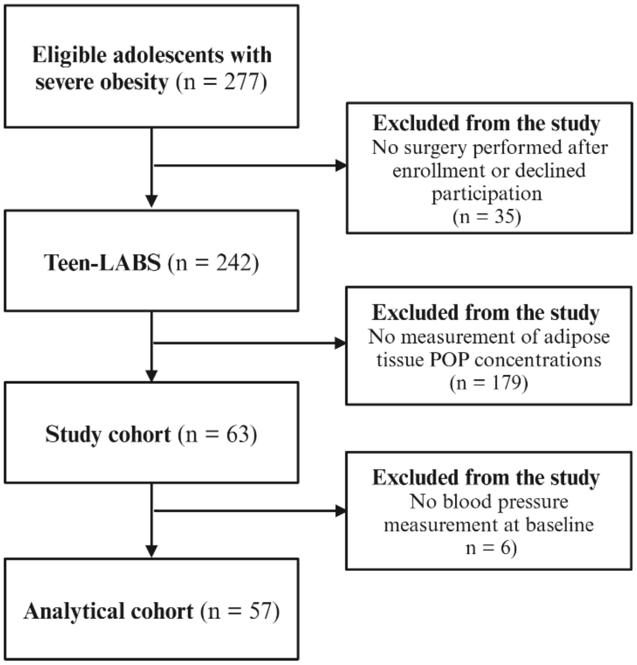

Figure S3: Population flowchart of the Teen-LABS cohort

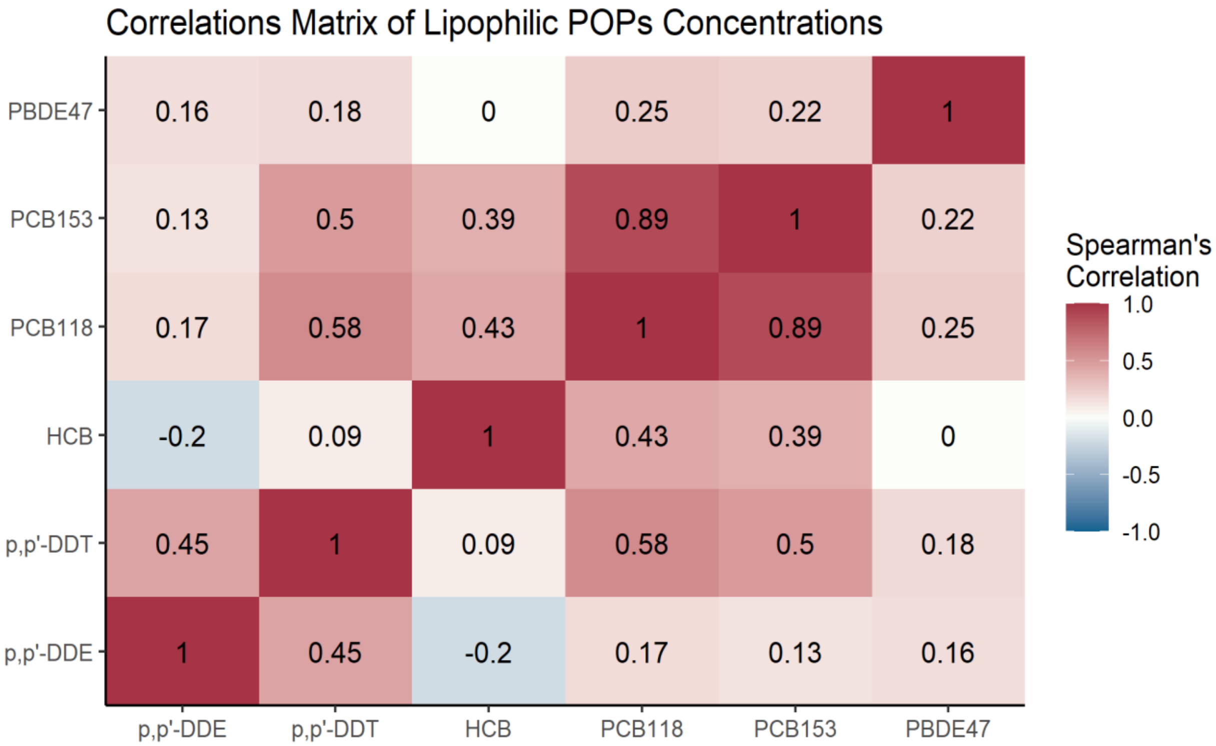

Figure S4: The Spearman's correlation between adipose tissue lipophilic POP mixtures.
